# Utility of Fine Needle Aspiration Supernatant for Real‐Time PCR–Based Mutation Analysis in Non–Small Cell Lung Cancer

**DOI:** 10.1002/dc.70184

**Published:** 2026-07-16

**Authors:** Adeyinka Akinsanya, Riley Gorden, Madison Rector, Hamna Muhammad, Melissa Randolph, Matt Sperling, Kemin Xu, Sheila Segura, Omer Saeed, Tieying Hou

**Affiliations:** ^1^ Department of Pathology and Laboratory Medicine Indiana University, School of Medicine Indianapolis Indiana USA

**Keywords:** collecting medium, fine needle aspiration, non‐small carcinoma lung cancer, real‐time PCR, supernatant

## Abstract

**Background:**

The emergence of personalized therapy and molecular sequencing has significantly transformed the management of advanced non–small cell lung carcinoma (NSCLC). Supernatants obtained from fine needle aspiration (FNA) have been shown to be a reliable source of high‐quality nucleic acid for molecular testing. One pre‐analytic factor that may influence the success of molecular analysis is the collection medium used for the FNA rinse.

**Methods:**

This prospective study evaluated 31 FNA specimens collected in three different media—CytoRich Red, 10% neutral buffered formalin, or 10% bovine serum albumin (BSA)—to assess their impact on DNA yield and real‐time polymerase chain reaction (RT‐PCR) outcomes for *KRAS (*Kirsten rat sarcoma viral oncogene homolog) and/or *EGFR* (epidermal growth factor receptor) mutations. DNA extraction was performed using the QIAamp DNA FFPE (formalin‐fixed paraffin‐embedded) Tissue Kit (Qiagen), and RT‐PCR was conducted using the QIAGEN therascreen *EGFR* or *KRAS* RGQ PCR kits. Concurrent direct smears or formalin‐fixed, paraffin‐embedded tissue from a cell block or core biopsy served as standard controls.

**Results:**

In the CytoRich Red group, 11 specimens (9 adenocarcinomas and 2 NSCLC) were analyzed. All 11 specimens generated results concordant with standard controls. One specimen required repeat DNA extraction due to initial pre‐test quality control failure. The formalin group included 12 specimens (7 squamous cell carcinomas [SCC], 3 adenocarcinomas, 1 NSCLC, and 1 large cell neuroendocrine carcinoma). Nine specimens produced concordant results with controls. Two specimens were negative for *EGFR* mutations, although the corresponding control tissues were not tested. Six required repeat DNA extraction because of initial pre‐test failure, and one specimen remained invalid after re‐extraction. Eight specimens were collected in BSA (5 adenocarcinomas, 2 NSCLC, and 1 SCC). Five produced concordant results with standard controls, while three specimens failed PCR pre‐tests despite repeat DNA extraction.

**Conclusion:**

In this small cohort, CytoRich Red demonstrated reliable performance for RT‐PCR‐based mutation testing. FNA supernatant represents a practical alternative specimen for lung cancer molecular analysis and may optimize specimen utilization while preserving cell block material.

## 1 Introduction

In the era of personalized medicine, molecular analysis of advanced non–small cell lung carcinoma (NSCLC), particularly adenocarcinoma, is imperative for identifying biomarkers that guide targeted therapies. In the latest version of the National Comprehensive Cancer Network (NCCN) Clinical Practice Guidelines in Oncology for NSCLC, the list of recommended molecular targets has expanded to include *EGFR* (epidermal growth factor receptor), *KRAS* (Kirsten rat sarcoma viral oncogene homolog), *ALK* (anaplastic lymphoma kinase), *ROS1* (ROS proto‐oncogene 1, receptor tyrosine kinase), *BRAF* (B‐Raf proto‐oncogene, serine/threonine kinase), *MET* (MET proto‐oncogene, receptor tyrosine kinase), *RET* (rearranged during transfection proto‐oncogene), *ERBB2* (Erb‐B2 receptor tyrosine kinase 2), *NTRK* (neurotrophic tropomyosin receptor kinase), and immunostains for programmed death‐ligand 1 (PD‐L1) and human epidermal growth factor receptor 2 (Her2) [1]. As the demand for molecular and ancillary testing increases, pathologists are frequently challenged to prioritize and optimize limited tissue samples for a broad range of analyses.

Endobronchial ultrasound–guided fine needle aspiration (EBUS‐FNA) has become the standard of care for evaluating patients with suspected lung cancer, as it is a minimally invasive approach that allows for both diagnosis and disease staging. In 2018, the College of American Pathologists, the International Association for the Study of Lung Cancer, and the Association for Molecular Pathology published updated consensus guidelines for molecular testing in lung cancer. These guidelines expanded recommendations for suitable cytology specimens from formalin‐fixed paraffin‐embedded cell blocks (FFPE CBs) to include any cytology specimens that have been validated within the institution [2].

Recent studies have demonstrated that supernatant fluid from FNA needle rinses yields high‐quality nucleic acid that can be successfully used to detect actionable mutations and gene fusions [3, 4, 5]. This application is not limited to lung cancer but has also been extended to other solid malignancies, such as melanoma [6] and thyroid carcinoma [7, 8, 9]. Use of supernatant fluid reduces the turnaround time from sample collection to arrival in the molecular laboratory by 3 to 5 days [4]. In addition, this approach preserves FFPE CBs material for cytomorphologic evaluation, immunohistochemistry, and other potential diagnostic and/or research applications.

A variety of collection media are currently used for FNA needle rinses across institutions. In this study, we evaluated three collection media: CytoRich Red, formalin, and bovine serum albumin (BSA), and assessed their impact on nucleic acid quality and molecular performance, using FFPE CBs, core biopsy, or direct smears as the reference standard.

## 2 Materials and Methods

After approval by the institutional review board, all FNA specimens were collected prospectively with rapid on‐site evaluation (ROSE) from 8/2024 to 4/2025. When the preliminary ROSE diagnosis indicated NSCLC, one designated needle pass was collected into 5 mL of one of three collection media—CytoRich Red, 10% neutral buffered formalin, or 10% BSA—for research purposes (Figure 1). Direct smears were prepared and stained with both Diff‐Quik (DQ) and Papanicolaou stains. Additional needle passes were collected in CytoRich Red for CBs preparation by using the HistoGel method. When a concurrent core biopsy was obtained, the specimen was fixed in 10% neutral buffered formalin. Following paraffin embedding, 4‐μm–thick sections were cut and stained with hematoxylin and eosin for both CBs and core biopsy specimens.

**FIGURE 1:**
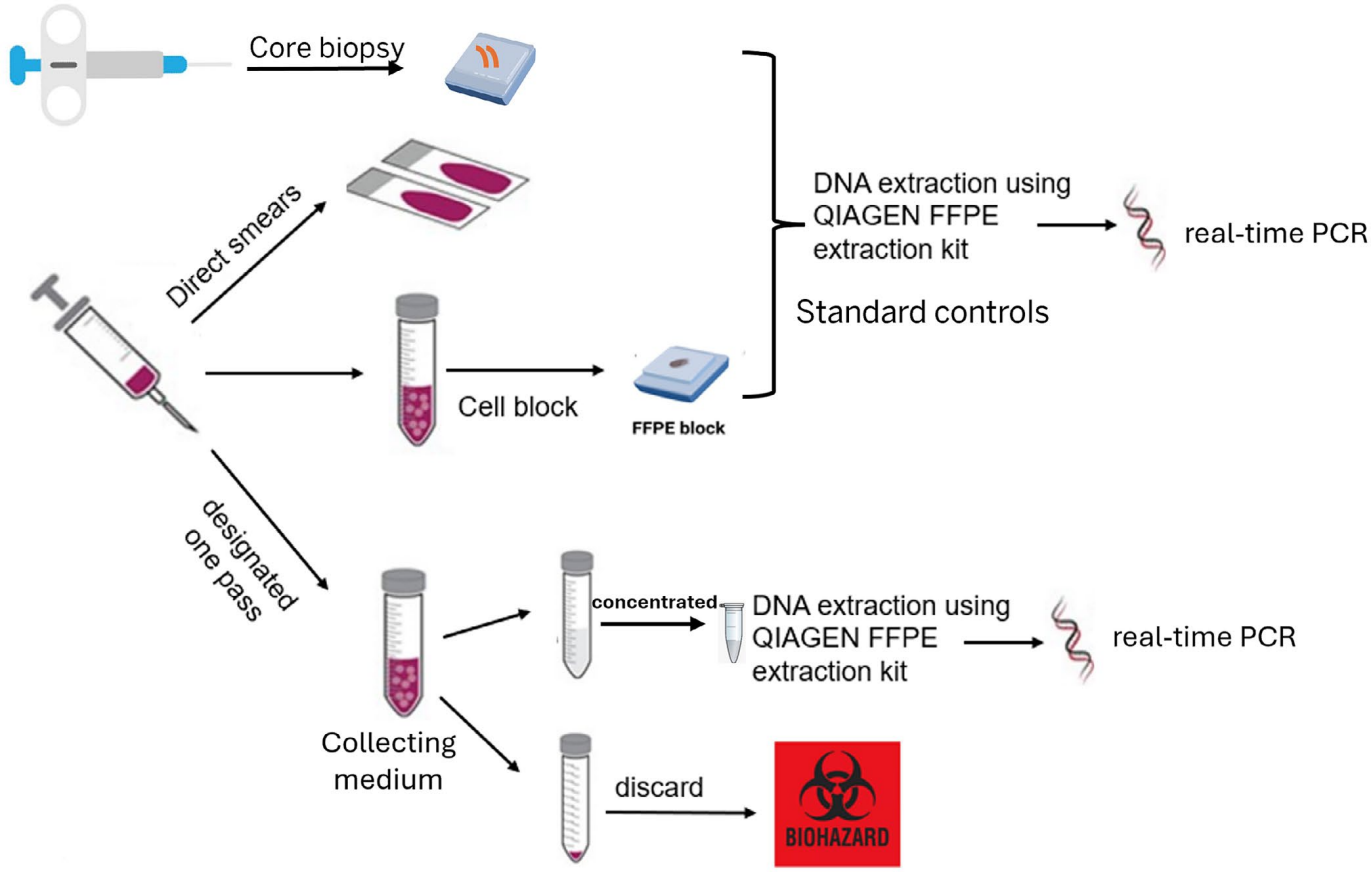
Workflow of cytology and core biopsy specimens for molecular testing. [Color figure can be viewed at wileyonlinelibrary.com]

Specimens collected in one of the three media were centrifuged at 3000 rpm for 5 min (Figure 1). To avoid interference with the routine specimen process for clinical diagnosis, the pellet from this research pass was discarded. The supernatant was transferred to a new tube and further concentrated by an additional centrifugation at 3000 rpm for 5 min. Approximately 2 mL of the concentrated sample was collected from each specimen and stored at −20°C until DNA extraction. One mL of the concentrated supernatant was used for DNA extraction using the QIAamp DNA FFPE Tissue Kit (Qiagen, Hilden, Germany). Following extraction, DNA concentration was measured using a NanoDrop spectrophotometer (Thermo Fisher Scientific, Waltham, MA, USA), and the extracted DNA was stored at −80°C until further analysis.

Corresponding standard control specimens underwent DNA extraction from DQ‐stained direct smears, CBs or core biopsy using the same kit and protocol. RT‐PCR‐based assays were performed to detect hotspot mutations in *KRAS* or *EGFR*, using Qiagen therascreen Rotor‐Gene Q (RGQ) kits [ARMS (Amplification Refractory Mutation System) and Scorpions real‐time polymerase chain reaction (RT‐PCR)]. Before doing mutation‐specific PCRs, the pre‐test (quality control step to evaluate total amplifiable DNA) was performed for both assays.

## 3 Results

A total of 11 specimens were collected in CytoRich Red, including 6 from lung lesions, 4 from mediastinal lymph nodes, and 1 from the chest wall (Table 1). Nine specimens were diagnosed as adenocarcinoma and 2 as NSCLC. The maximum interval between FNA specimen collection and DNA extraction was 94 days. DNA yields ranged from 132 to 3804 ng. *KRAS* mutation analysis was performed in 10 specimens, while both *KRAS* and *EGFR* mutation analyses were conducted in one case. *KRAS* mutations were identified in 5 specimens, including 2 p.G12C, 2 p.G12V, and 1 p.G13D mutations. *EGFR* mutation was negative in case 11. All supernatant specimens showed 100% concordance with their corresponding standard controls.

**TABLE 1:** Mutation analysis results from specimens collected in CytoRich Red.

ID	Source	FNA Dx	DNA yield (ng)	Repeat extraction	Mutation tested	Results	Standard controls	Concordant
C1	Lung	AdenoCA	192	Yes	*KRAS*	p.G12V	Smears	Yes
C2	Lung	NSCLC	348	No	*KRAS*	Neg	Smears	Yes
C3	Lung	AdenoCA	1248	No	*KRAS*	Neg	Cell block	Yes
C4	LN*	AdenoCA	840	No	*KRAS*	Neg	Smears	Yes
C5	LN	NSCLC	264	No	*KRAS*	p.G12C	Smears	Yes
C6	Lung	AdenoCA	468	No	*KRAS*	Neg	Core biopsy	Yes
C7	LN	AdenoCA	252	No	*KRAS*	p.G13D	Smears	Yes
C8	Lung	AdenoCA	168	No	*KRAS*	p.G12C	Core biopsy	Yes
C9	LN	AdenoCA	132	No	*KRAS*	Neg	Cell block	Yes
C10	Chest wall	AdenoCA	204	Yes	*KRAS*	p.G12V	Cell block	Yes
C11	Lung	AdenoCA	3804	No	*KRAS/EGFR*	Neg	Smears	Yes

Abbreviations: AdenoCA: adenocarcinoma; Dx: Diagnosis; LN: lymph nodes; Neg: negative; NSCLC: non‐small cell carcinoma.

* All LNs were from mediastinal lymph nodes.

Twelve specimens were collected in 10% neutral buffered formalin, including 10 from lung tumors and 2 from mediastinal lymph nodes (Table 2). This cohort included 7 squamous cell carcinomas (SCC), 3 adenocarcinomas, 1 NSCLC, and 1 large cell neuroendocrine carcinoma (LCNEC). The FNA supernatant was stored at −20°C for up to 136 days before DNA extraction. The total DNA yield ranged from 72 to 1248 ng. *EGFR* mutation analysis was performed in all 7 SCC specimens, while *KRAS* mutation analysis was performed in the remaining 5 cases. Valid results were obtained in 11 of 12 tested specimens, including 3 *KRAS*‐mutant cases (2 with p.G12A and 1 p.G12C) and 8 negative cases (2 *KRAS* negative, 6 *EGFR* negative). Nine of these 11 specimens showed concordance with corresponding controls. In the remaining 2 SCC cases, molecular testing was not performed in the control group. Six of the 12 specimens required repeat DNA extraction due to failed pre‐test PCR sequencing runs, including 2 adenocarcinomas and 4 SCCs. One specimen failed the PCR pre‐testing despite repeat DNA extraction.

**TABLE 2:** Mutation analysis results from specimens collected in 10% neutral buffer formalin.

ID	Source	FNA Dx	DNA yield (ng)	Repeat extraction	Mutation tested	Results	Standard controls	Concordant
F1	Lung	AdenoCA	1248	No	*KRAS*	p.G12A	Smears	Yes
F2	Lung	AdenoCA	300	Yes	*KRAS*	p.G12A	Smears	Yes
F3	LN*	NSCLC	624	Yes	*KRAS*	Neg	Cell block	Yes
F4	Lung	AdenoCA	144	No	*KRAS*	p.G12C	Smears	Yes
F5	Lung	LCNEC	72	No	*KRAS*	Neg	Smears	Yes
F6	Lung	SCC	144	No	*EGFR*	Neg	Cell block	Yes
F7	Lung	SCC	1152	No	*EGFR*	Neg	Cell block	Yes
F8	Lung	SCC	312	No	*EGFR*	Neg	Core biopsy	Yes
F9	LN	SCC	1140	Yes	*EGFR*	Neg	Not tested	N/A
F10	Lung	SCC	396	Yes	*EGFR*	Neg	Core biopsy	Yes
F11	Lung	SCC	168	Yes	*EGFR*	Neg	Not tested	N/A
F12	Lung	SCC	1176	Yes	*EGFR*	Invalid	Not tested	N/A

Abbreviations: AdenoCA: adenocarcinoma; Dx: diagnosis; LN: lymph nodes; N/A: not applicable; Neg: negative; NSCLC: non‐small cell carcinoma.

* All LNs were from mediastinal lymph nodes.

Eight specimens were collected in 10% BSA, including 5 from mediastinal lymph nodes and 3 from lung tumors (Table 3). The cohort included 5 adenocarcinomas, 2 NSCLC, and 1 SCC. The storage time for the supernatant was up to 120 days. DNA production ranged from 120 to 420 ng. *KRAS* mutation analysis was performed in 6 cases, *EGFR* mutation analysis in 1 case, and both *KRAS* and *EGFR* analyses in 1 case. Valid results were obtained in 5 of the 8 specimens. All were negative for mutations and were fully concordant with the corresponding controls. PCR pre‐testing was unsuccessful in the remaining 3 specimens despite repeat DNA extraction.

**TABLE 3:** Mutation analysis results from specimens collected in 10% BSA.

ID	Source	FNA Dx	DNA yield (ng)	Repeat extraction	Mutation tested	Results	Standard controls	Concordant
B1	LN*	SCC	420	No	*KRAS/EGFR*	Neg	Cell block	Yes
B2	LN	NSCLC	384	No	*EGFR*	Neg	Smears	Yes
B3	LN	AdenoCA	204	No	*KRAS*	Neg	Smears	Yes
B4	LN	AdenoCA	120	No	*KRAS*	Neg	Cell block	Yes
B5	Lung	AdenoCA	168	No	*KRAS*	Neg	Smears	Yes
B6	Lung	NSCLC	216	Yes	*KRAS*	Invalid	Smears	Control Neg
B7	LN	AdenoCA	204	Yes	*KRAS*	Invalid	Cell block	Control Neg
B8	Lung	AdenoCA	156	Yes	*KRAS*	Invalid	Smears	Control Neg

Abbreviations: AdenoCA: adenocarcinoma; Dx: diagnosis; LN: lymph nodes; N/A: not applicable; Neg: negative; NSCLC: non‐small cell carcinoma.

* All LNs were from mediastinal lymph nodes.

## 4 Discussion

Molecular testing is essential in guiding the treatment of advanced NSCLC. In our current practice, RT‐PCR‐based mutation analysis is performed for *EGFR*, *KRAS*, and *BRAF* mutations using direct smears, while immunohistochemical staining for PD‐L1 and ALK is performed on FFPE CBs or core biopsies. FISH studies for *ROS1* rearrangement and reflex *ALK* rearrangement, when indicated, are also assessed on FFPE specimens. After completion of these studies, there is often very limited residual material available for additional testing. Consequently, further molecular analysis by next generation sequencing (NGS) is frequently deferred to surgical resections. The FNA supernatant, which is typically discarded during specimen processing, has been shown to provide high‐quality nucleic acids suitable for molecular testing [3, 4, 10, 11, 12, 13, 14, 15, 16]. During validation of this approach, we observed that various collection media and processing protocols have been reported in the literature (Table S1) [3, 4, 10, 11, 12, 13, 14, 15, 16, 17]. The collection media used include CytoLyt [3, 4, 10, 16], normal saline [3, 12], RPMI 1640 [13, 17], or formalin [11]. All of these media have been demonstrated to be compatible with downstream molecular analysis.

In this study, we evaluated three different collection media—CytoRich Red, formalin, and BSA—as part of our validation process. For all three media, specimens that passed the pre‐test quality control step yielded 100% concordance with the standard controls. However, 2 specimens from CytoRich Red, 6 specimens from formalin, and 3 specimens from the BSA group failed the quality control pre‐test. Following repeated DNA extraction, 1 specimen in formalin and 3 supernatants from the BSA group remained invalid in the pre‐test. We observed that the majority of these cases were collected at satellite hospitals, requiring transportation of specimens at room temperature to the main lab for processing. Nucleic acid degradation during transport could be a potential cause. BSA was selected as one of the collection media because it has been shown to bind PCR inhibitors and improve enzyme performance [18]. However, it may not be suitable for long‐term preservation. We suspect that the specimens which failed the pre‐test in the BSA medium may have degraded due to the absence of fixation during storage. In this context, the use of non‐fixative media such as BSA is not recommended for molecular testing workflows, particularly when specimens may require delayed processing or transport between facilities.

Formalin has been shown to cause cross‐linking between nucleic acids and proteins, leading to DNA fragmentation and reduced polymerase accessibility [19, 20]. In contrast, non–cross‐linking, alcohol‐based cytology fixation media generally yield higher‐quality DNA and provide superior performance for molecular testing. Compared with CytoLyt, CytoRich Red contains a small amount of formaldehyde, which enhances long‐term preservation. At the start of our project, we were concerned that this formaldehyde component might negatively affect PCR results. However, in this small cohort, CytoRich Red proved to be a reliable medium for RT‐PCR. Supernatants stored at −20°C for up to 94 days consistently yielded sufficient DNA, and all specimens produced results concordant with the corresponding controls.

Discarded supernatants from FNA preparation not only provide an alternative source for molecular testing, but also optimize workflow by reducing turnaround time and cost. Gokozan et al. [10] reported that NGS performed on supernatant reduced turnaround time by approximately 30% and resulted in a cost savings of $50 per sample. The time savings were primarily attributed to elimination of steps required for FFPE processing, including retrieval of blocks, sectioning of unstained slides, assessment of adequacy, and deparaffinization during DNA extraction. In a recent study, Maher et al. [13] demonstrated that fully automated nucleic acid extraction using the Genexus Integrated System further reduced hands‐on time, enabling completion of sequencing and data analysis within 24 h of specimen collection without compromising performance.

Another important consideration is how to integrate supernatant collection for molecular testing into the overall workflow in a way that still allows appropriate use of ancillary studies such as immunohistochemical stains (IHC). Alcohol‐based fixation used in cytology specimen preparation has been shown to affect PD‐L1 assessment in NSCLC. Multiple studies have evaluated cytology–histology concordance for PD‐L1 scoring and, overall, have demonstrated that cytology specimens are feasible for PD‐L1 evaluation [21]; however, reported concordance rates vary widely, ranging from approximately 50% to 100% [21]. This variability is likely influenced by multiple factors, including specimen cellularity, the reference specimen used for comparison, fixation medium, cell block preparation methods, the PD‐L1 antibody clone used, and tumor heterogeneity. A recent study showed that [22], compared with PreservCyt and CytoLyt, CytoRich Red demonstrated better concordance with formalin‐fixed cell blocks across all three PD‐L1 antibody clones (28–8, 22C3, and SP263). Nevertheless, all alcohol‐based fixatives produced significantly weaker PD‐L1 staining intensity than formalin, regardless of antibody, raising the risk of underestimating PD‐L1 expression and false‐negative results, particularly at the ≥ 1% cutoff [22]. Similarly, Pineda et al. [23] reported overall lower HER2 IHC scores in CytoLyt‐fixed cell blocks compared with surgical pathology specimens. With the expanding panel of lung cancer biomarkers, including emerging immunostains such as c‐MET [24] and B7‐H3 [25], careful validation of each antibody is necessary to ensure reliable performance in cell block material.

One limitation of this study is that the prospective design did not allow selection of specific tumor types. Adenocarcinoma specimens are generally preferred because comprehensive molecular panels are routinely performed for these cases, allowing them to serve as positive controls. However, during specimen collection for the formalin group, a higher proportion of SCC cases was encountered. This uneven distribution of tumor types among the three groups may have introduced bias into the results. Second, due to budgetary constraints, the sample size in each group was limited, and only one or two genes were tested per sample. It is challenging to draw definitive conclusions due to the small cohort size, particularly when comparing CytoRich Red with formalin collection medium. At the time of manuscript preparation, our institution is transitioning to an NGS‐based companion diagnostic panel encompassing 22 genes. Therefore, validation of FNA supernatant for this NGS panel is planned in the near future.

## 5 Conclusions

Despite variability in collection media and processing protocols, EBUS‐FNA supernatant consistently yielded high‐quality nucleic acids and showed high concordance with reference standard molecular results. Incorporating supernatants into routine workflows may improve specimen utilization, particularly in small or limited samples. Our findings also suggest that CytoRich Red is a suitable collection medium for downstream RT‐PCR–based mutation analysis.

## Author Contributions

Design, data analysis, manuscript draft, table, and figures: A.A., T.H., M.R.; technical support: R.G., C.M., H.M., M.R., M.S.; manuscript editing: K.X., S.S., O.S.

## Funding

This work was supported by the Department of Pathology and Laboratory Medicine, Indiana U.

## Disclosure

The authors have nothing to report.

## Conflicts of Interest

The authors declare no conflicts of interest.

## Supporting information


**Table S1:** Summary of literature using supernatants from FNA of lung cancer for molecular tests.

## Data Availability

The data that support the findings of this study are available from the corresponding author upon reasonable request.
