# Supplementary material for: Utility of Fine Needle Aspiration Supernatant for Real‐Time PCR–Based Mutation Analysis in Non–Small Cell Lung Cancer
Source: Diagn Cytopathol. 2026 Jul 16;54(11):805–11. doi: 10.1002/dc.70184 (PMC13633391; doi:10.1002/dc.70184)
Supplement: Supplementary file 1 [file DC-54-805-s001.docx]

| **Journal** | **Authors** | **Sample size** | **Collecting medium** | **Storage temperature** | **Storage days** | **Extraction platform/kit** | **Molecular test** | **Successful rate** | **Standard controls** | **Concordant rate** |
| --- | --- | --- | --- | --- | --- | --- | --- | --- | --- | --- |
| Lung Cancer, 2018 ^3^ | Guibert, N, et al | 17 | CytoLyt or saline | -80℃ | unknown | QIAamp circulating nucleic-acid kit | NGS, ddPCR | 100% for NGS | CB | 100% |
| Cancer Cytopathol, 2019 ^4^ | Janaki, N, et al | 30 | CytoLyt | 4℃ | 7 to 29 | Maxell RSC DNA and RNA kits (Promega) | NGS | 100% | CB | 100% |
| Ann Oncol, 2019 ^13^ | Hannigan, B, et al | 116 | RPMI 1640 | 4℃ | unknown | Qiagen circulating DNA kit, QIAsymphony automatic DNA  extraction platform | NGS, ddPCR | 90% | CB  CNB  Plasma | 100%  96%  84% |
| Diagn Cytopathol, 2020 ^6^ | Gokozan, H, et al | 62 | CytoLyt | 4℃ | Up to 30 | unknown | NGS | unknown | CB | unknown |
| Cancer Cytopathol, 2020 ^12^ | Wu, W, et al | 214 | CytoLyt | -20℃ | unknown | QIAamp circulating nucleic acid kits circulating DNA kits (AmoyDx) | Super-ARMS *EGFR*  detection kits (AmoyDx) | 100% | CB | 97.2% |
| Diagn Cytopathol, 2021 ^10^ | Tafoya, M, et al | 23 | unknown | 5℃ | 3-41 | Puregene method  (Qiagen) | NGS | 87% | CB  CNB | 100% |
| Thorac Cancer, 2022 ^8^ | Kong, F, et al | 48 | Normal saline | -20℃ | unknown | QIAamp Circulating Nucleic Acid Kit | RT-PCR | 100% | CNB | 95.8% |
| Cancer Cytopathol, 2024 ^9^ | Maher, M, et al | 50 | RPMI 1640 | -80℃ | unknown | The MagMax Cell‐Free Total Nucleic Acid Isolation Kit, the Genexus Cell‐Free Total Nucleic Acid Purification Kit | NGS | 97% | CNB  Plasma | 100% |
| J Am Soc Cytopathol, 2024 ^7^ | Jager, L, et al | 5 | formalin | 4℃ | unknown | MagMAX Cell-Free Total Nucleic Acid Isolation Kit | NGS | 100% | CB | 100% |
| Pulmonology, 2025 ^11^ | Rodrigues, LV, et al | 15 | unknown | unknown | unknown | unknown | NGS | 100% | CB | 100% |

**Supplemental Table 1:** Summary of literature using supernatants from FNA of lung cancer for molecular tests

FNA: fine needle aspiration; NGS: next generation sequencing; ddPCR: digital droplet PCR; RT-PCT: real time PCR; CB: cell block; CNB: core needle biopsy
